# Differences and significance of peripheral blood interleukin-6 expression between patients with granulomatous lobular mastitis and those with benign breast tumors

**DOI:** 10.3389/fmed.2023.1273406

**Published:** 2023-09-25

**Authors:** Yue Zhou, Jingjing Wu, Lina Ma, Bing Wang, Tian Meng, Hongfeng Chen, Meina Ye

**Affiliations:** Department of Breast Surgery (Traditional), Longhua Hospital Affiliated to Shanghai University of Traditional Chinese Medicine, Shanghai, China

**Keywords:** cancer-associated inflammatory cytokines, interleukin-6 (IL-6), breast benign tumor, granulomatous lobular mastitis, correlation analysis

## Abstract

**Objective:**

It is unclear whether the mechanism of the interleukin (IL)-6 signaling pathway is similar between granulomatous lobular mastitis (GLM) and benign breast tumors. This study aimed to explore the differences and significance of peripheral blood IL-6 and related cytokines, routine blood test results, and C-reactive protein (CRP) levels between patients with GLM and benign breast tumors.

**Methods:**

Seventy-three inpatients with GLM who underwent surgery and 60 patients with benign breast tumors diagnosed based on pathological findings between November 2022 and May 2023 were included. The white blood cell (WBC) and neutrophil (NEU) counts were determined using an automatic blood cell analyzer, the CRP level was determined by an immunoturbidimetric assay, and serum IL-6 and related cytokine levels were determined by an enzyme-linked immunosorbent assay.

**Results:**

The WBC, NEU, and CRP values in patients with GLM were significantly higher than those in patients with benign breast tumors (*P* < 0.01). Serum IL-6 levels were significantly higher in patients with GLM than in those with benign breast tumors (*P* < 0.01). There were no significant differences in the serum concentrations of IL-1β, IL-7, and interferon (IFN)-γ between patients with GLM and those with benign breast tumors (*P* > 0.05), but the tumor necrosis factor (TNF)-α level was higher in patients with GLM than in those with benign breast tumors (*P* < 0.01). In patients with GLM, the Pearson correlation analysis showed that the IL-6 level was positively correlated with NEU, NEU%, CRP, IL-17, and TNF-α values (*P* < 0.01). Additionally, the IL-6 level was weakly positively correlated with WBC and IFN-γ values. Conversely, in patients with benign breast tumors, the IL-6 level was not significantly correlated with the aforementioned indicators in routine blood tests but was positively correlated with IL-17, IFN-γ, and TNF-α values (*P* < 0.01).

**Conclusions:**

IL-6, NEU, NEU%, and CRP values were significantly elevated in patients with GLM compared to those with benign breast tumors, indicating that IL-6 plays an important role in the development and onset of GLM. The correlation between these cytokines and the development and progression of benign breast tumors needs to be further explored, as cytokines such as IL-6 may provide effective markers for the treatment of GLM.

## 1. Introduction

Granulomatous lobular mastitis (GLM) is a rare, chronic, non-specific inflammatory disease of the breast that has seen an increasing incidence in recent years (1). Although GLM is an inflammatory disease, its clinical characteristics are complex and diverse, especially at the mass stage, making it difficult to distinguish between benign breast tumors and breast cancer. The etiology of this disease remains unclear. However, antibiotics and hormones have poor effects on GLM. It easily suppurates and ulcerates repeatedly, forming sinuses, fistulas, or ulcers, thus resulting in breast disfiguration, which seriously affects the patient's quality of life (2). This disease is difficult to treat clinically, and its clinical and radiographic features can simulate breast cancer. Current clinical studies have suggested that the pathogenesis of GLM includes inflammation, immune reactions, apoptosis, tissue damage, and gene polymorphisms. However, its etiology remains controversial (3). Therefore, it is of great importance and value to explore the etiology and pathogenesis of GLM.

Interleukin (IL)-6 is a pleiotropic cytokine that is both an inflammatory cytokine and a cancer-associated cytokine (4). In an inflammatory tumor microenvironment, IL-6 has demonstrated the ability to induce pro- and anti-inflammatory responses using three mechanisms of signal transduction (classical signaling, transsignaling, and cluster signaling), interact with a diversity of target cells, and induce endocrine effects in an autocrine/paracrine manner (5). Studies have shown that the IL-6/Janus tyrosine family kinase (JAK)/signal transducer and the activator of the transcription 3 (STAT3) signaling pathway is closely related to plasma cell mastitis (6, 7) and idiopathic granulomatous mastitis (8). However, the in-depth pathogenesis of GLM has not been reported. Existing studies have confirmed that the IL-6/JAK/STAT3 signaling pathway is crucial for the proliferation and survival of tumor cells (9); however, it is unclear whether this pathway plays a similar role in GLM formation and whether its mechanism is similar to that in benign breast tumor. No studies have investigated whether the IL-6 signaling pathway is involved in the formation of GLM. In our previous study, we quantitatively analyzed the difference in protein expression in GLM lesion tissues and healthy breast tissues adjacent to the lesion using tandem mass tag (TMT) quantitative proteomics technology and found that the expression of the STAT3 protein in GLM lesion tissues was significantly higher than that in healthy breast tissues. Based on this finding, this study aimed to further explore the differences and significance of IL-6 in the development of GLM and benign breast tumors to provide new ideas and bases for clinical diagnosis and treatment.

## 2. Materials and methods

### 2.1. Patients

This study included 73 operative inpatients with GLM and 60 operative inpatients with benign breast tumors (excluding breast cysts or benign breast tumors with inflammation) from the Department of Breast Surgery (Traditional), Longhua Hospital Affiliated to Shanghai University of Traditional Chinese Medicine, who were diagnosed based on pathological findings between November 2022 and May 2023.

### 2.2. Laboratory data

This study was a retrospective trial. Serum samples were collected from patients with GLM and those with benign breast tumors. A total of 5 ml of fasting venous blood was obtained from each patient on the day of hospital admission, placed in an ethylenediaminetetraacetic acid anticoagulant tube, and sent to the Clinic Laboratory of Longhua Hospital within 2 h for routine blood testing and for determining the C-reactive protein (CRP) and cytokine levels. The white blood cell (WBC) count, neutrophil (NEU) count, and neutrophilic granulocyte percentage (NEU %) were detected using an automatic hematology analyzer. CRP was detected by immunoturbidimetry, and serum cytokines were detected by an enzyme-linked immunosorbent assay.

### 2.3. Statistical analysis

Continuous variables were reported as mean (±standard deviation). The independent *t*-test was performed to analyze normally distributed values, and the Pearson's method was employed to perform correlation analysis. To predict the IL-6 levels in patients with GLM, the logistic regression model was employed, and the independent variables of time since the appearance of masses (≤3 months = 0, >3 months = 1), mass size (<10 cm = 0, ≥10 cm = 1), antibiotic use, and glucocorticoid use for treating this disease were integrated. SPSS software (version 27.0; IBM Corp.) was used to analyze all data. All the *p*-values reported were two-sided, and *p*-values < 0.05 were considered statistically significant.

## 3. Results

### 3.1. Patient characteristics

Clinical characteristics of the 133 inpatients are shown in Table 1. Specific clinical characteristics of patients with benign breast tumors and those with GLM are presented in Tables 2, 3, respectively. All patients were women with an average age of 32.90 ± 5.67 (range, 20–45) years. The average ages of patients with GLM and those with benign breast tumors were 33.38 ± 4.86 years and 32.32 ± 6.52 years, respectively, without a significant difference between the two groups (*P* > 0.05). None of the patients had immune system diseases, and they were not treated with immunosuppressants.

**Table 1:** Clinical characteristics of the 133 inpatients.

**Characteristics**		**Group**	
		**Granulomatous lobular mastitis (*****n** =* **73)**	**Benign breast tumor (*****n** =* **60)**
Age, years, mean (SD)		33.38 (4.86)	32.32 (6.52)
BMI, mean (SD)		23.56 (4.2)	21.25 (2.39)
Childbearing history, *N* (%)	o	1 (1.4%)	24 (40.0%)
	1	60 (82.2%)	30 (50.0%)
	2	12 (16.4%)	6 (10.0%)
Elevated prolactin level, *N* (%)		24 (32.9%)	6 (10.0%)
Elevated cholesterol level, *N* (%)		19 (26.0%)	18 (30.0%)
Elevated triglycerides level, *N* (%)		14 (19.2%)	2 (3.3%)
Breast surgery history, *N* (%)		5 (6.8%)	7 (11.7%)

**Table 2:** Clinical characteristics of the 60 inpatients with benign breast tumors.

**Characteristics**		***N* (%)**
Pattern	Unilateral	25 (41.7%)
	Bilateral	35 (58.3%)
Number of tumors	1	12 (20%)
	2–5	36 (60%)
	≥5	12 (20%)
Time since tumors appearing	< 1 year	23 (38.3%)
	1–5 years	28 (46.7%)
	≥5 years	9 (15.0%)
Breast imaging reporting and data system (BI-RADS)	Unknown	2 (3.3%)
	3	44 (73.3%)
	3–4a	9 (15.0%)
	4a	5 (8.3%)
Family history of breast tumors	Benign tumor	2 (3.3%)
	Breast cancer	1 (1.7%)

**Table 3:** Clinical characteristics of the 73 inpatients with granulomatous lobular mastitis.

**Characteristics**		***N* (%)**
Pattern	Unilateral	67 (91.8%)
	Bilateral	6 (8.2%)
Time since the appearance of lumps	< 1 month	2 (2.7%)
	1–3 months	27 (40.0%)
	>3 months	44 (60.3%)
Size of the lumps (palpation)	< 5 cm	2 (2.7%)
	5–10 cm	44 (60.3%)
	≥10 cm	27 (37.0%)
Extent of lesions (MRI)	≤ 2 quadrants	42 (57.5%)
	>2 quadrants	31 (42.5%)
Lesions involving the areola		69 (94.5%)
Nipple inversion		26 (35.6%)
Erythema nodosum		17 (23.3%)
Antibiotic use		37 (50.1%)
Glucocorticoid use		12 (16.4%)
History of GLM		5 (6.8%)

### 3.2. Comparisons of routine blood test results and the CRP level

Based on routine blood tests of the 73 patients with GLM, the average WBC count was 7.91 ± 2.31 × 10^9^/L and 17 of the patients (23.3%) had a higher WBC count than the upper limit of normal value (9.5 × 10^9^/L). The average NEU count was 5.64 ± 2.03 × 10^9^/L; 22 patients (30.1%) had an NEU count higher than the upper limit of normal value (6.3 × 10^9^/L). The average NEU% was 68.70 ± 13.23%; 41 patients (56.2%) had an NEU% higher than the upper limit of normal value (70%). The average CRP level was 4.45 ± 5.85 mg/L; 19 patients (24%) had a CRP level higher than the upper limit of normal value (5 mg/L).

Based on routine blood tests of the 60 patients with breast benign tumors, the average WBC count was 5.75 ± 1.42 × 10^9^/L. One of the patients (1.7%) had a WBC count higher than the upper limit of normal value. The average NEU count was 3.69 ± 1.38 × 10^9^/L; 3 patients (5%) had an NEU count higher than the upper limit of normal value. The average NEU% was 62.76 ± 9.91%; 12 patients (20.0%) had an NEU% higher than the upper limit of normal value. The average CRP level was 0.49 ± 0.50 mg/L; no patients had a CRP level higher than the upper limit of normal value.

**Figure 1:**
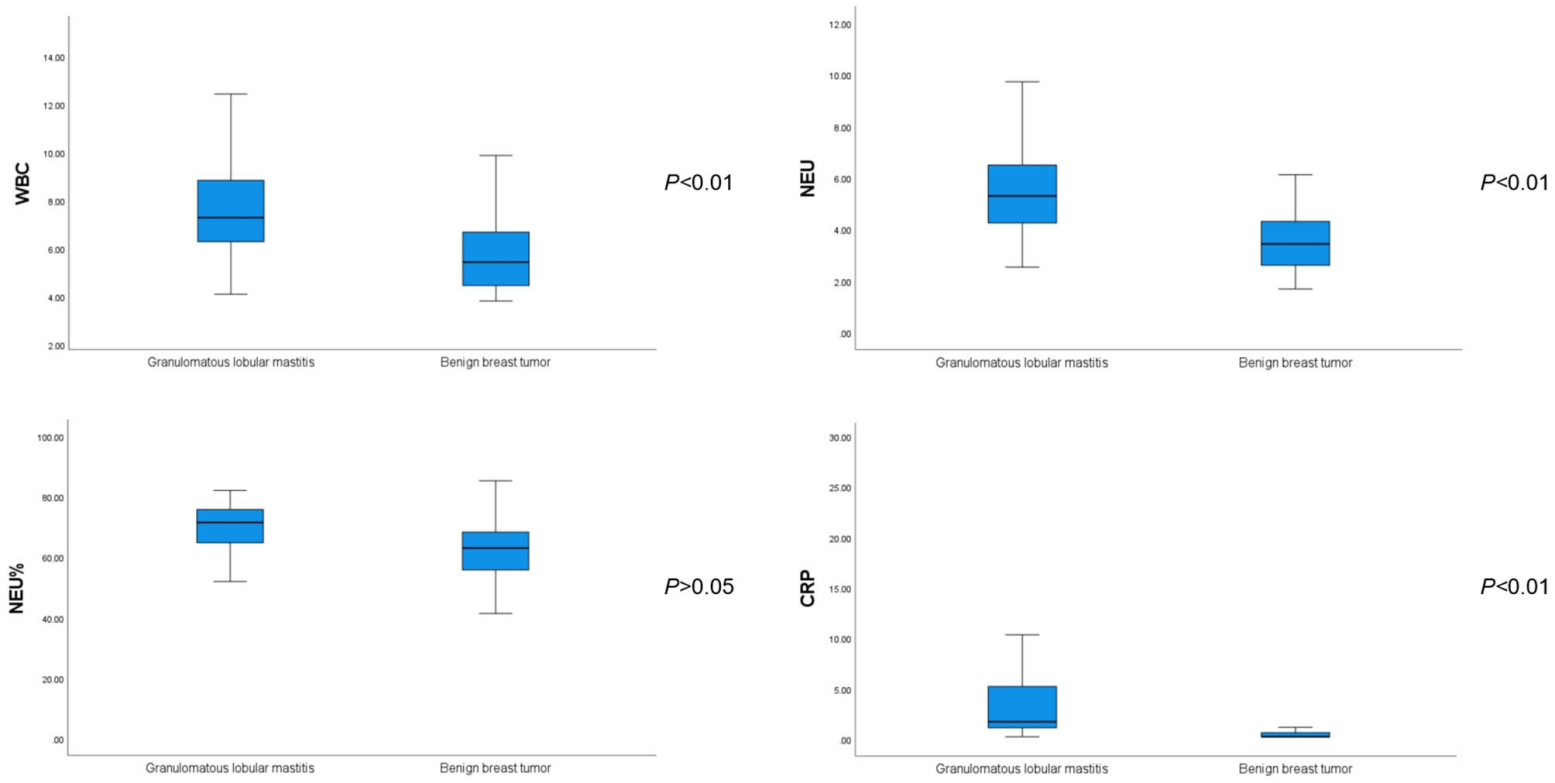
Comparison of white blood cell, neutrophil, neutrophilic granulocyte percentage, and C-reactive protein values between patients with GLM and those with benign breast tumors.

The WBC, NEU, and CRP values were significantly higher in patients with GLM than in those with benign breast tumors (*P* < 0.01). However, there was no significant difference in the NEU% between the groups (*P* > 0.05), as shown in Table 4 and Figure 1.

**Table 4:** Comparison of routine blood test results and CRP levels between patients with GLM and benign breast tumors ($\bar{X}\pm s$).

**Variable**		**WBC ( × 10^9^/L)**	**NEU ( × 10^9^/L)**	**NEU% (%)**	**CRP (mg/L)**
**Group**	GLM (*n =* 73)	7.91 ± 2.31	5.64 ± 2.03	68.70 ± 13.23	4.45 ± 5.85
	Benign tumor (*n =* 60)	5.75 ± 1.42	3.69 ± 1.38	62.76 ± 9.91	0.49 ± 0.50
***P*** **-value**		0.001	0.009	0.966	<0.001

### 3.3. Comparisons of serum cytokine levels

**Figure 2:**
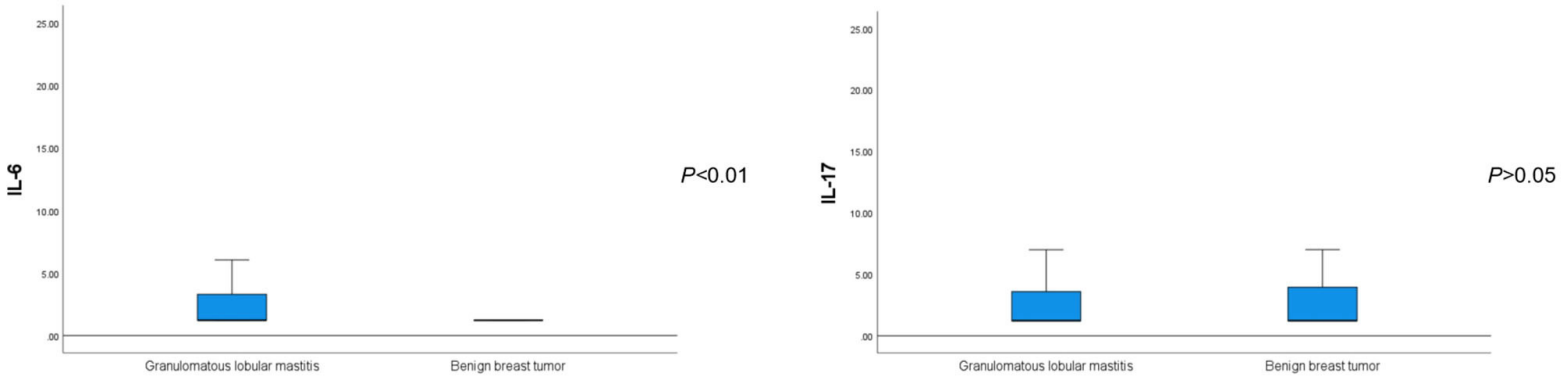
Comparison of serum IL-6 and IL-17 levels between patients with GLM and those with benign breast tumors.

The average serum IL-6 concentration in patients with GLM was 2.99 ± 3.34 pg/ml, which was significantly higher than that in patients with benign breast tumors (*P* < 0.01). Regarding other inflammatory and tumor-associated cytokines, there was no significant difference in the serum IL-17 concentration between patients with GLM and those with benign breast tumors (*P* > 0.05) (Figure 2). IL-β1 and IFN-γ concentrations were not significantly different between the two groups (*P* < 0.1), whereas serum TNF-α concentrations in patients with GLM were significantly higher than those in patients with benign breast tumors (*P* < 0.01), as shown in Table 5.

**Table 5:** Comparison of serum cytokine levels between patients with GLM and those with benign breast tumors ($\bar{X}\pm s$).

**Variable**		**IL-6 (pg/ml)**	**IL-1β (pg/ml)**	**IL-17 (pg/ml)**	**IFN-γ (pg/ml)**	**TNF-α (pg/ml)**
**Group**	GLM (*n =* 73)	2.99 ± 3.35	3.30 ± 4.53	6.91 ± 9.57	2.89 ± 2.10	1.69 ± 1.74
	Benign tumor (*n =* 60)	1.25 ± 0.20	2.66 ± 2.17	5.46 ± 5.78	2.26 ± 1.71	1.36 ± 0.65
***P*** **-value**		< 0.001	0.097	0.108	0.053	0.009

### 3.4. Correlation analysis of IL-6 levels and inflammatory indicators

In patients with GLM, the Pearson correlation analysis showed that IL-6 levels were positively correlated with NEU, NEU%, CRP, IL-17, and TNF-γ values (*P* < 0.01) but weakly positively correlated with WBC and IFN-γ values (*P* < 0.1). In patients with benign breast tumors, the IL-6 level was not significantly correlated with the aforementioned indicators in routine blood tests (*P* > 0.05), but it was positively correlated with IL-17, TNF-α, and IFN-γ levels (*P* < 0.01), as shown in Tables 6, 7, respectively.

**Table 6:** Correlation analysis between IL-6 levels and inflammatory indicators in patients with GLM.

**Variable**	**WBC**		**NEU**		**NEU%**		**CRP**		**IL-1**β		**IL-17**		**IFN-**γ		**TNF-**α	
**IL-6**	***r*** **-value**	***P*** **-value**	***r*** **-value**	***P*** **-value**	***r*** **-value**	***P*** **-value**	***r*** **-value**	***P*** **-value**	***r*** **-value**	***P*** **-value**	***r*** **-value**	***P*** **-value**	***r*** **-value**	***P*** **-value**	***r*** **-value**	***P*** **-value**
	0.212	0.071	0.305	0.009	0.302	0.009	0.506	< 0.001	0.147	0.215	0.300	0.010	0.204	0.084	0.325	0.005

**Table 7:** Correlation analysis between IL-6 and inflammatory indicators in patients with benign breast tumor.

**Variable**	**WBC**		**NEU**		**NEU%**		**CRP**		**IL-1**β		**IL-17**		**IFN-**γ		**TNF-**α	
**IL-6**	***r*** **-value**	***P*** **-value**	***r*** **-value**	***P*** **-value**	***r*** **-value**	***P*** **-value**	***r*** **-value**	***P*** **-value**	***r*** **-value**	***P*** **-value**	***r*** **-value**	***P*** **-value**	***r*** **-value**	***P*** **-value**	***r*** **-value**	***P*** **-value**
	0.011	0.935	0.004	0.974	0.011	0.933	−0.062	0.636	−0.096	0.464	0.377	0.003	0.372	0.003	0.673	< 0.001

### 3.5. Logistic regression analysis of factors associated with IL-6 levels in patients with GLM

**Figure 3:**
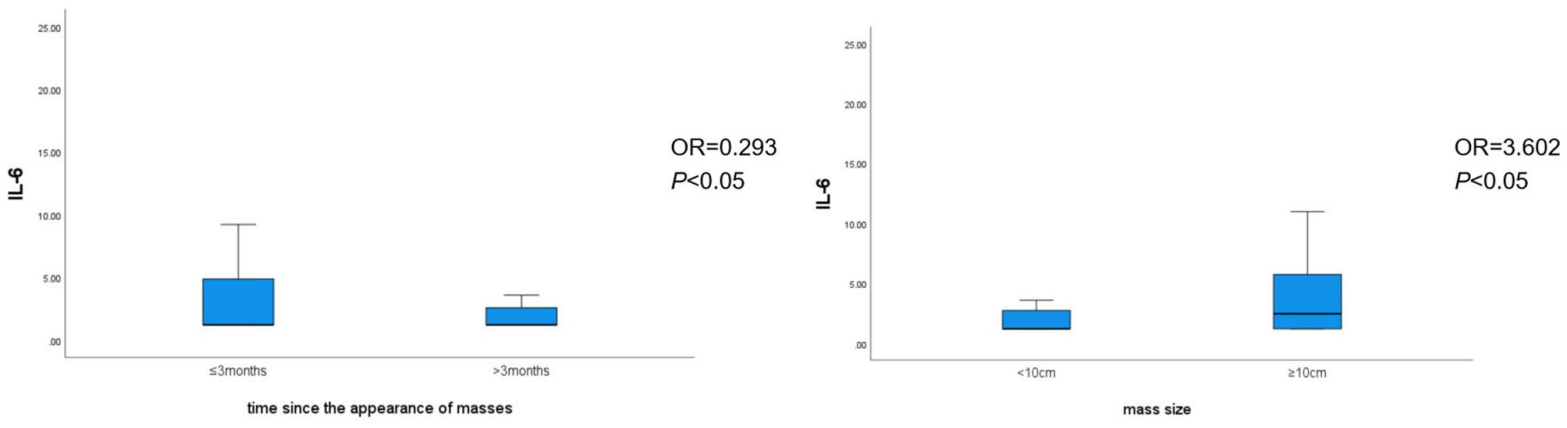
Correlation between the time since the appearance of masses and mass size with IL-6 levels.

Binary logistic regression analysis revealed that the time since the appearance of masses [odds ratio (OR) = 0.293, *P* < 0.05), mass size (OR = 3.602, *P* < 0.05), and glucocorticoid use (OR = 5.690, *P* < 0.05) were individual predictors of IL-6 levels in patients with GLM (Table 8). The correlation between the time since the appearance of masses and mass size with IL-6 levels is shown in Figure 3.

**Table 8:** Logistic regression analysis of factors associated with IL-6 levels in patients with GLM.

	**β-value**	***P*-value**	**O*R*-value**	**95% CI**
Time since the appearance of masses	−1.228	0.033	0.293	0.095~0.906
Mass size (palpation)	1.282	0.024	3.602	1.179~11.004
Antibiotics use	0.427	0.437	1.533	0.522~4.505
Glucocorticoids use	1.739	0.017	5.690	1.163~27.838

## 4. Discussion

### 4.1. GLM is a rare, chronic, non-specific inflammatory disease

GLM mainly involves the mammary lobules and is characterized by non-caseating granulomas centered on the mammary lobules (10). It is an independent and peculiar disease of the breast that, although benign, is insensitive to routine anti-inflammatory therapy and may relapse even after repeated surgical treatments. Since a breast mass is the most common initial symptom, GLM is difficult to diagnose clinically in the early stage of the disease and is easily misdiagnosed as a benign or malignant breast tumor. If misdiagnosed as a tumor, it cannot be treated with appropriate methods at the right time, resulting in the spread and protracted course of the disease (11). Therefore, it poses a challenge in the breast surgery field.

In addition, the etiology and pathogenesis of GLM are unclear, although internal experts and those abroad have unanimously recognized the following as etiologies: (1) Deposit and blockage of the lactiferous duct by laticifer secretions: Breast duct obstruction and secretion accumulation can develop for various reasons, and then, the congested substances can stimulate the duct wall, causing the wall to produce inflammatory cells and promote fibroplasia, resulting in intense inflammatory reactions and (2) autoimmune reactions caused by the overflow of substances in the duct: Breast duct obstruction can cause duct dilation, which, to a certain extent, can lead to atrophy of the epithelium of the duct wall, and the accumulation of lipids and epithelial cell debris can corrode the duct wall, resulting in duct wall damage and thereby leading to autoimmune response (12).

Most scholars believe that GLM is an aseptic inflammatory disease, whereas others believe that it is related to infections caused by specific bacteria. However, the positivity rate in bacterial cultures of GLM samples is low; therefore, its pathogenesis is controversial (13). Clinical studies have shown that inflammatory indicators, such as the WBC count, CRP level, and erythrocyte sedimentation rate; autoimmune indicators, such as lymphocytes, immunoglobulin, and antinuclear antibody profiles; and endocrine indicators, such as prolactin, which are related to the disease, are less significant in making a definite diagnosis (14). Similarly, in this study, we observed that the proportion of patients with hyperprolactinemia was higher in the GLM group (32.9%) than in the benign breast tumor group (10.0%; *P* < 0.01). Interestingly, the body mass index was higher in patients with GLM than in those with benign breast tumors (*P* < 0.05), suggesting that obesity may be related to the pathogenesis of GLM.

In our previous study, we quantitatively analyzed the difference in protein expression in GLM lesion tissues and healthy breast tissues adjacent to the lesion using TMT quantitative proteomics technology and found that the expression of STAT3 protein in GLM lesion tissues was significantly higher than that in healthy breast tissues. In the present study, we further investigated the differences in the peripheral blood inflammatory indicator IL-6 and other cytokines in patients with GLM and those with benign breast tumors, which provided an innovative idea for exploring the pathogenesis, clinical diagnosis, and treatment of GLM.

### 4.2. Role of IL-6 in inflammatory reactions and the tumor microenvironment

IL-6 is an inflammatory cytokine that is closely related to inflammatory reactions and is a cancer-associated inflammatory factor. Pleiotropic cytokine TNF-α produced by macrophages, endothelial cells, and smooth muscle cells can stimulate smooth muscle cells to produce IL-6, together with IL-β1 and IFN-γ. IL-6 is a central regulator of inflammatory reactions that can stimulate the release of vasoactive substances and induce fibrinogen secretion and CRP production (15). The involvement of IL-6 in the pathophysiology of inflammatory diseases makes it an important target for the treatment of these diseases (16). In the tumor microenvironment, IL-6 can be produced by various cells, including tumor-associated macrophages, granulocytes, and tumor cells, which are its main sources. T-cells and myeloid-derived suppressor cells can also contribute to elevated IL-6 levels in tumors (17). IL-6 is released by tumor and stromal cells in an autocrine or paracrine manner and infiltrates the local area of solid tumors. The inhibition and activation of IL-6 may be involved in the development and progression of tumors by regulating the expression of inflammatory cytokines. They can promote tumor cell proliferation and inhibit apoptosis through transduction of the JAK signal and transcription of the STAT (18, 19), phosphoinositol 3-kinase, mitogen-activated protein kinase, and extracellular signal-regulated kinase 1/2 pathways, altering the tumor microenvironment and the immune response and thus affecting the growth, invasion, and metastasis of tumors (20). Recent studies have compiled evidence indicating that the IL-6 cytokine family (soluble factors) may be used for early and more precise breast cancer diagnosis and for designing targeted therapy to treat or even prevent metastasis development (21).

STAT3 signal transduction, mediated by IL-6, is an important signaling pathway that has recently been discovered. This pathway, stimulated and activated by cytokines, plays a crucial role in many important biological processes such as cell proliferation, differentiation, apoptosis, and immune response regulation (22). As an important intracellular signaling pathway, the IL-6/JAK2/ STAT3 pathway plays an important role in cell proliferation and differentiation by affecting the activation status of various downstream effector molecules. Excessive release of IL-6 under inflammatory stimulation is an effective activator of the JAK2/STAT3 signaling pathway, and IL-6 may promote the process of epithelial-mesenchymal transition by activating this pathway to exert its pro-inflammatory effect (23). In the tumor microenvironment, continuous stimulation with IL-6 can activate JAK2, resulting in STAT3 phosphorylation and thereby promoting tumor growth, drug resistance, and metastasis. Activation of the IL-6/JAK2/STAT3 signaling pathway is involved in the development and progression of tumors and contributes to the formation of the tumor inflammatory microenvironment (24).

Although some studies have shown that the IL-6/JAK2/STAT3 signaling pathway is related to non-puerperal mastitis, such reports are few. Our previous study showed that STAT3 protein expression was significantly higher in GLM lesion tissues than in healthy breast tissues. As the STAT3 protein is a key transcriptional regulatory factor in the IL-6 signaling pathway, whether this finding suggests that IL-6 is involved in the formation of GLM and benign breast tumors and whether there is a difference in the roles of IL-6 in these two diseases were the main objectives of the present study.

### 4.3. Difference and significance of peripheral blood IL-6 expression in patients with GLM and those with benign breast tumors

It is difficult to explain the poor effects of antibiotics and glucocorticoids in treating GLM. Few studies have examined the association between biomarkers and clinical outcomes. In this study, we observed that WBC, NEU, and CRP values were higher in patients with GLM than in those with benign breast tumors, but WBC, NEU, NEU%, and CRP values were not elevated in most patients with GLM, which explains the poor effect of antibiotics and glucocorticoids in the treatment of this disease.

It is of great importance to examine whether serum IL-6 expression differs between patients with GLM and those with benign breast tumors and to explore the correlation between IL-6 and traditional inflammatory indicators, such as WBC, NRU, and CRP values, as GLM is an inflammatory disease and IL-6 is a pleiotropic cytokine. This study's results showed that the serum IL-6 level was significantly higher in patients with GLM than in those with benign breast tumors. Concerning the other inflammatory and tumor-associated cytokines, the serum TNF-α level was significantly higher in patients with GLM than in those with benign breast tumors; IL-β1 and IFN-γ levels were slightly higher in patients with GLM than in those with benign breast tumors; and elevation of the IL-17 level did not show any significant difference between the two diseases. Additionally, binary logistic regression analysis revealed that serum IL-6 levels may be elevated early because of the presence of breast masses (within <3 months) and large masses (>1 cm).

In patients with GLM, further correlation analysis showed that the IL-6 level was positively correlated with NEU, NEU%, CRP, IL-17, and TNF-α values, but it was weakly positively correlated with WBC and IFN-γ values. In contrast, in patients with benign breast tumors, the IL-6 level was not significantly correlated with the aforementioned indicators in routine blood tests but was positively correlated with cytokines IL-17, IFN-γ, and TNF-α. The correlation between IL-6 and TNF-α was more significant in patients with benign breast tumors than in those with GLM patients, which is worth noting and requires further research.

## 5. Conclusions

Serum levels of IL-6, TNF-α, and other inflammatory and cancer-associated cytokines, as well as WBC, NEU, and CRP values, have a certain correlation with the development and progression of GLM; thus, they can reflect the disease to a certain extent. However, current treatments for GLM, such as surgery and antibiotic and glucocorticoid, immunosuppressant, and prolactin inhibitor therapies, are ineffective and lack validation.

Therefore, it is hoped that the exploration of cytokines such as IL-6 can provide effective markers for the treatment of GLM. With further research, over-activation of the STAT3 protein is receiving more attention, and increasing evidence suggests that the IL-6/STAT3 signaling pathway plays an important role in the inflammation, development, and progression of tumors. Specific interventions targeting the related proteins and enzymes in this pathway may provide new ideas for the treatment of inflammation and tumors. It is also hoped that this signaling pathway can serve as a reference for the investigation of the mechanism and design of drugs, thereby becoming one of the directions for the study of treatment for GLM.

## Data availability statement

The datasets presented in this study can be found in online repositories. The names of the repository/repositories and accession number(s) can be found in the article/supplementary material.

## Ethics statement

The studies involving humans were approved by the Longhua Hospital Affiliated to Shanghai University of Traditional Chinese Medicine. The studies were conducted in accordance with the local legislation and institutional requirements. The participants provided their written informed consent to participate in this study.

## Author contributions

HC: Supervision, Writing—review and editing. MY: Funding acquisition, Writing—review and editing. YZ: Funding acquisition, Writing—original draft, Writing—review and editing. JW: Resources, Writing—review and editing, Supervision. LM: Data curation, Writing—review and editing. BW: Methodology, Writing—review and editing. TM: Methodology, Writing—review and editing.
